# Near-infrared responsive theranostic platforms: toxicological challenges, biological interactions, and clinical translation

**DOI:** 10.3389/fbioe.2026.1998995

**Published:** 2026-10-28

**Authors:** Vivek S. S. Chougule, Sweta Acharya, Adarsh Sharma, Arya Ojha, Ankit Jain

**Affiliations:** Industrial Research Laboratory, Department of Pharmacy, Birla Institute of Technology and Science Pilani, Pilani, India

**Keywords:** biodistribution, disease microenvironment, nanomedicine, near-infrared, photodynamic therapy, photothermal therapy, theranostic platforms

## Abstract

The disease microenvironment is a complex milieu containing unique biochemical and biophysical cues, such as acidosis, oxidative stress, hypoxia, enzymatic imbalance, and microorganisms, which influence not only the effectiveness of treatments but also the fate and safety of the nanomedicine. NIR-responsive dyes and dye-conjugated nanomaterials have attracted considerable attention for diagnostic and therapeutic applications owing to their deep tissue penetration, external-stimulus activation, and precise spatiotemporal control. Despite notable progress in developing NIR-responsive systems for cancer, inflammatory diseases, and infections, their clinical use is limited by issues such as toxicity, degradation, biodistribution, accumulation, persistence, and immune reactions to nanomaterials. Importantly, recent studies have shown that the disease microenvironment can significantly affect the degradation, activation, bioactivity, and toxicity of NIR-responsive systems, yet these issues remain scattered in the literature. Specific attention is paid to structure–toxicity relationships, phototoxicity, immunotoxicity, biodegradability, biodistribution, organ-specific accumulation, and long-term safety, as well as emerging contrasts between NIR-I and NIR-II-based systems. We additionally elaborate on advanced experimental models, regulatory approaches, and safety and design approaches, such as biodegradable systems, renal-clearance-based systems, and smart activated nanomaterials.

## 1 Introduction

Near-Infrared (NIR) responsive theranostic platforms have emerged as among the most promising approaches in precision medicine by combining diagnosis and treatment within a single multifunctional platform (Li and Pu, 2019; Liu et al., 2019). The special characteristics of NIR radiation, including its ability to penetrate deep into tissues, low scattering by photons, and negligible tissue autofluorescence, make it possible to perform non-invasive imaging and highly targeted therapy that overcomes numerous problems of traditional treatments (Hong et al., 2017). As a result, there has been increasing interest in the use of NIR-responsive nanomaterials for cancer, infections, inflammation, cardiovascular disease, and neurodegenerative disease applications (Shetty et al., 2019). Another key aspect that is responsible for the efficacy of NIR-responsive nanomedicine is the existence of disease-specific microenvironments that differ from healthy ones (Lu et al., 2016; Hanahan and Weinberg, 2011). The pathological tissues usually show low pH levels of the extracellular environment, hypoxia, elevated levels of reactive oxygen species (ROS), increased vascular permeability, increased formation of extracellular matrix, changes in the expression of enzymes, and an inflammatory response (Lu et al., 2016; Hanahan and Weinberg, 2011). Exploitation of these unique features of the microenvironment has enabled the development of stimuli-responsive nanocarriers that can selectively release drugs, improve imaging capabilities, and enable the localization of photothermal and photodynamic treatments, thus enhancing efficacy and reducing toxicity (Zhou Q. et al., 2023).

The rapid development of nanotechnology has led to the emergence of many near-infrared-responsive dyes and nanoparticles with enhanced optical efficiency and multifunctionality (Li et al., 2024). Organic fluorophores such as cyanine dyes, squaraine dyes, diketopyrrolopyrroles, BODIPY derivatives, and conjugated polymers have been found to exhibit desirable biodegradation characteristics and controllable optical properties, albeit with photobleaching constraints (Lovell et al., 2024). However, inorganic nanomaterials such as gold nanostructures, copper sulfide nanoparticles, transition-metal dichalcogenides, quantum dots, rare-earth nanoparticles, and carbon nanomaterials offer high efficiency in photothermal energy conversion and imaging but pose challenges related to long-term tissue retention, biodegradation, and metal toxicity (Duan et al., 2023). However, due to certain drawbacks of organic and inorganic nanoparticles, hybrid nanoplatforms comprising both organic and inorganic materials have been designed to enhance stability and therapeutic outcomes while reducing adverse effects (Altinbasak et al., 2024). Despite significant advances in preclinical studies, the successful translation of such therapeutic systems to the clinic has been hindered by unresolved safety and toxicological issues (Joyce et al., 2024). The activity of the said nano-sized materials is governed not only by their physicochemical properties (e.g., the size, shape, charge, surface properties, chemical make-up of nanoparticles)but also by the way they behave in biological systems once injected (Almeida et al., 2024). After being administered to the body, the nanoparticles undergo a series of steps, beginning with the formation of the protein corona and culminating in elimination from the body, which affects their efficiency and safety (Barz et al., 2024; Cabral et al., 2024). It should be noted that a pathologically altered environment can profoundly affect the biological processes involving nanoparticles (Zhao et al., 2024). Therefore, thorough toxicological studies (including safety evaluations, biological interactions within the diseased microenvironment, and standard regulatory testing) are necessary for the nanoparticles to be used effectively in clinical practice (Havelikar et al., 2024). The present paper focuses on the safety and toxicity issues concerning the NIR dyes and dye-loaded nanoparticles, with special attention being paid to the impact of the pathological microenvironments on their performance. The pathological microenvironment affects the biological fate of NIR-responsive nanomaterials (Chen X. et al., 2024). Also, the review provides a critical analysis of the interrelationships among the structure and toxicity of nanomaterials, their modes of interaction with biological systems, phototoxicity and immunotoxicity, regulatory hurdles, and novel safety-by-design approaches, which can hasten the clinical translation of near-infrared (NIR)-active theranostics.

## 2 NIR-responsive nanomaterials for targeted therapy

The use of near-infrared (NIR) light is increasingly favored in theranostics due to its remarkable properties, including deep tissue penetration and low toxicity levels. Thanks to these advantages, it is possible to obtain accurate diagnostic imaging and activate therapy non-invasively by utilizing various types of nanomaterials whose characteristics depend on NIR wavelengths (Hossain et al., 2022; Raza et al., 2019). The near infrared range is divided into two parts–the first biological window–NIR-I (700–950 nm) and the second biological window–NIR-II (1000–1700 nm). NIR-I technology is popular for its availability of laser systems and approved fluorophores such as indocyanine green (ICG). However, NIR-II technology is preferred for its deeper penetration, lower photon scattering, and reduced background fluorescence (Reich, 2005; Bh et al., 2018).

NIR-responsive nanomaterials are classified into three types: organic dyes and nanomaterials, inorganic nanomaterials, and hybrid platforms. Organic systems such as ICG and cyanine dyes have tunable properties but suffer from stability and degradation issues. Inorganic systems such as gold nanostructures and carbon-based materials offer strong optical or photothermal responses but need toxicological assessment due to potential accumulation. Hybrid platforms combine both to enhance functions, yet their complexity raises concerns about toxicity, stability, and biological interactions. These categories help understand how composition affects optical performance, cellular interactions, degradation, biodistribution, and toxicity.

NIR-responsive nanomaterials operate via multiple mechanisms, one of which is photothermal conversion: the process by which they absorb light and convert it into heat in photothermal therapy (PTT). Another mechanism involves photodynamic activation, in which photosensitizers generate reactive oxygen species (ROS) during photodynamic therapy (PDT). Furthermore, NIR excitation allows for fluorescence and photoacoustic imaging, resulting in real-time therapeutic response tracking (Hossain et al., 2022; Wang et al., 2025). NIR-I has been prominent in clinical applications due to its well-established technology and approved imaging agents. At the same time, NIR-II represents the next-generation of the optical window, offering excellent imaging efficacy and therapeutic accuracy. However, more studies on biosafety, standardized instruments, and clinical validation are required for it to become widely utilized in clinical practice (Shanmugam et al., 2014; Tomar et al., 2025). NIR-responsive nanomaterials enable photothermal therapy (PTT), photodynamic therapy (PDT), fluorescence imaging, and photoacoustic imaging (PAI) through distinct photophysical mechanisms. Their applications span cancer, infectious diseases, cardiovascular and neurological disorders, and wound healing, with therapeutic and diagnostic outcomes depending on material composition and irradiation conditions. However, enhanced optical performance may also influence cellular toxicity, immune responses, tissue accumulation, and degradation. Therefore, understanding the relationship between material properties and biological safety is essential for evaluating the structure–toxicity relationships.

Nanomaterials responsive to NIR can serve as theranostics combining diagnostics and therapy into a single nanosystem. They have been studied for cancer (Belia et al., 2022), microbial infections (Li et al., 2026), cardiovascular (Rauschendorfer et al., 2024), and neurological diseases (Mansour et al., 2024), wounds (Yao et al., 2025), and inflammatory conditions (Yao et al., 2025), in which environmental factors such as hypoxia, acidic pH, ROS, glutathione, and enzymes are present. Both NIR-I (650–950 nm) and NIR-II (1000–1700 nm) induce fluorescence or photoacoustic signals, enabling PTT, PDT, drug release, and imaging-guided therapies such as ablation, chemotherapy, immunotherapy, bacterial imaging, and antimicrobial treatments (Figure 1). They are relevant for vascular inflammation, thrombosis, neuroinflammation, ischemia, and wounds. Benefits of combining NIR imaging with therapy are offset by challenges such as tissue penetration, safety, oxygen dependence, biocompatibility, distribution, and clinical translation (Zhang S. et al., 2025).

## 3 Structure and toxicity relationships of NIR-responsive nanomaterials

The safety and performance of NIR-responsive nanomaterials depend on factors like material composition, physicochemical properties, surface characteristics, biological interactions, and degradation. Understanding these is crucial for safety-by-design. Factors such as dye chemistry, size, shape, surface charge, functionalization, stability, and decomposition affect interactions with proteins, membranes, organelles, and barriers. Their biological activity results from nanomaterial and NIR irradiation, which induce heating, chemical reactions, ROS generation, and decomposition. Toxicity varies with structure, dose, irradiation target, environment, and metabolism. Differences in size, surface chemistry, and coating influence cellular uptake, distribution, elimination, and toxicity (Liu et al., 2016).

### 3.1 Influence of NIR-Active dye chemistry

The optical and photobiological features of near-infrared-active chromophores depend on their molecular structure. Modifying these structures through π-conjugation length, heterocyclic rings, or groups such as electron donors/acceptors and terminal groups in dyes such as cyanines, ICG, BODIPY, squaraines, and porphyrins can control absorption and emission in NIR-I and NIR-II. These modifications affect excitation energy, fluorescence, internal conversion, intersystem crossing, and non-radiative relaxation. In phototheranostics, increased non-radiative relaxation enhances thermal action, while efficient intersystem crossing generates triplet states and ROS, thereby influencing photodynamic effects. Structural changes intended to improve NIR properties could reduce safety. Recent studies show minor chemical changes impact ROS production, cellular absorption, and light toxicity; halogen substitution in cyanines affects ROS, toxicity, absorption, and phototoxicity in carboxylic analogs (Pontremoli et al., 2023; Yang et al., 2026).

Only some cyanines showed significant light-dependent cytotoxicity, emphasizing the role of molecular structure in biological response (Delaey et al., 2000) (Figure 2).

**FIGURE 1:**
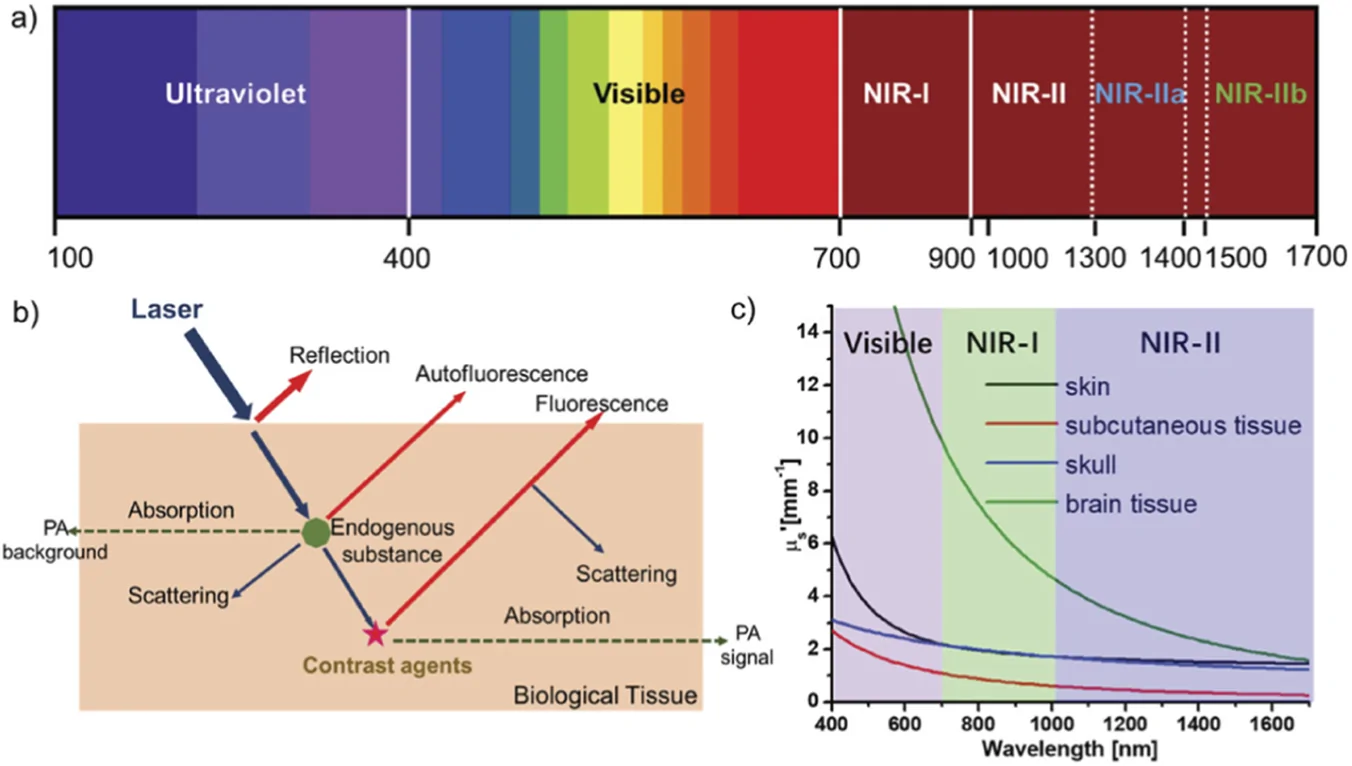
**(a)** Wavelengths for fluorescence imaging and PAI. **(b)** Schematic illustration of light–tissue interaction. **(c)** The reduced scattering coefficient, µs, is plotted as a function of wavelength in the range of 400–1700 nm for various tissue types. Reproduced with permission from (Ding et al., 2019) Copyright © 2019 WILEY‐VCH Verlag GmbH & Co. KGaA, Weinheim.

**FIGURE 2:**
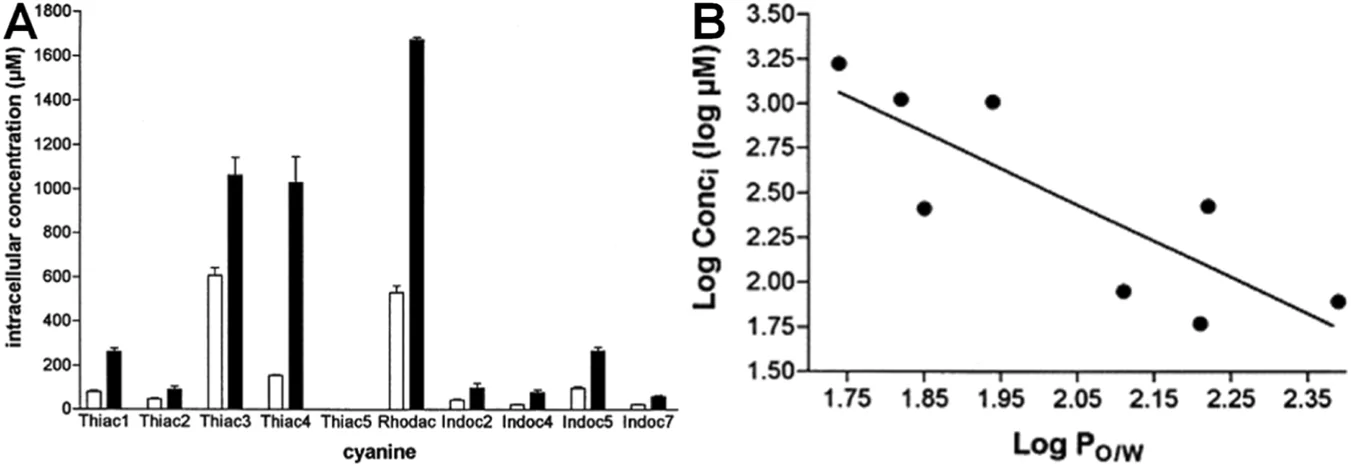
**(A)** The intracellular concentration of the selected cyanines in HeLa cells. The cells were incubated in the dark with 1 μM (open bars) and 5 μM (filled bars) for 24 h. Bars represent the mean ± SD of different experiments (*n* = 3), and **(B)** the logarithm of the intracellular concentration (log Conc_i_) of some selected cyanines in HeLa cells, after a 24 h incubation with 5 μM, as a function of the logarithm of the partition coefficient (log *P*_O/W_). Two compounds (Thiac5 and Indoc2) were excluded from this analysis. Reproduced with permission from (Delaey et al., 2000), Copyright © 2000 Elsevier Science S.A. All rights reserved.

Polymethine chromophore design influences aggregation, photostability, solvatochromism, protein interactions, and *in vivo* behavior. While extending π-conjugation shifts absorption into the NIR-II range, highly conjugated polymethines risk aggregation, quenching, photodegradation, and altered biomolecular interactions. Nanoformulation helps by controlling particle size, shielding, and pharmacokinetics, but does not replace toxicity assessment of the dye, its metabolites, and breakdown products (Ran et al., 2026; Xiang et al., 2026).

Dye chemistry’s importance spans photochemical stability and photoinduced products. Under prolonged irradiation, NIR chromophores may oxidize or cleave, altering biological properties. Structural changes can reduce ROS for photothermal use or increase ROS for PDT.

### 3.2 Influence of nanomaterial composition

Incorporating the NIR-active component into a nanoscale carrier or mixing it with an NIR-absorbing material makes the nanomaterial’s composition a key factor in its toxicity. NIR-responsive nanoplatforms include gold nanostructures, copper chalcogenides, transition-metal oxides, lanthanide-doped upconversion nanoparticles (UCNPs), quantum dots, carbon materials, conjugated polymers, organic nanoparticles, silica systems, and organic–inorganic hybrids. These vary in stability, reactivity, dissolution, biodegradability, ion release, protein adsorption, and clearance. Consequently, two nanomaterials with similar NIR absorption or photothermal efficiency can have quite different toxicity profiles, especially inorganic photothermal materials, where persistence and incomplete clearance raise safety concerns despite good optical performance (He et al., 2016; Abbasi et al., 2023).

NIR absorbers based on noble metals are not necessarily biologically inert, as toxicity can stem from surface ligands, synthesis reagents, coatings, or physicochemical interactions. Gold nanorods exemplify this: despite their inert gold core, their synthesis often uses CTAB, a surfactant that forms a positively charged layer around the nanorods. Studies show that the toxicity of CTAB-capped gold nanorods primarily arises from CTAB, whereas polymer coatings reduce toxicity. CTAB-coated nanorods can induce mitochondrial damage, ROS, apoptosis, and autophagy, regardless of aspect ratio, whereas coatings such as polystyrene sulfonate reduce these effects. These results highlight that surface corona composition can be more toxicologically relevant than the core itself (Alkilany et al., 2009).

Semiconducting and metal-chalcogenide NIR absorbers may cause toxicity through dissolution or ion release. Copper sulfide and copper-deficient chalcogenides are widely used for NIR photothermal applications due to their absorption and heat-conversion properties. Their biological fate depends on copper release and metabolism. CuS nanoplates exhibit dose-dependent toxicity in endothelial and macrophage cells. Intravenous injection primarily results in accumulation in the liver, spleen, and lungs (Feng et al., 2015). Recent studies show that the size and composition of biomineralized Cu_2_-xS nanoparticles affect their distribution and toxicity: small particles disperse and are fecally cleared, whereas larger ones accumulate in the liver, causing hepatotoxicity. Optimizing NIR-responsive nanomaterials involves considering optical efficiency, stability, ion release, organ retention, metabolism, and long-term toxicity. This is crucial for UCNPs, in which the host lattice, dopants, ligands, and coatings influence biological behavior. These factors influence stability, protein interactions, cellular uptake, and toxicity, leading to ROS production, membrane disruption, oxidative stress, DNA damage, mitochondrial issues, and inflammation. UCNP toxicity depends on composition, size, surface charge, shape, coating, dose, and exposure duration (Khan et al., 2025). Therefore, the compositional effects described above naturally converge with the physical parameters of nanomaterial, particularly size, morphology, and surface characteristics.

### 3.3 Role of nanomaterial morphology, size, and surface charge

The composition’s influence pertains to the nanomaterial’s 3D structure and interfacial properties, including size, shape, aspect ratio, curvature, and surface charge, which collectively affect the nano–bio interface. These factors influence protein adsorption, corona formation, membrane interaction, endocytosis, trafficking, tissue penetration, recognition, clearance, and organ retention. For NIR materials, morphology impacts optical resonance and electromagnetic fields; increasing the aspect ratio of gold nanorods shifts the LSPR into NIR. Morphology optimization concerns optical and toxicological effects. Smaller particles have increased surface area, reactivity, cellular interactions, tissue penetration, and renal clearance if exceedingly small. The size-toxicity relationship is not always linear; some studies show that very small, metabolizable gold nanorods retain NIR properties with lower retention and better clearance. Seven-nanometer gold nanorods had a lower residual body burden than 14-nm AuNRs after 30 days. Conversely, smaller nanoparticles can be more reactive due to a larger surface area and intracellular access. Size optimization should consider optical performance, target-site accumulation, cellular uptake, and elimination, rather than assuming smaller or larger particles are inherently safer (Feng et al., 2015; Li et al., 2016; Gnach et al., 2015).

Morphology’s impact on toxicity is independent of chemical structure. Shapes like rods, spheres, stars, cubes, and bipyramids have different features such as surface area, curvature, activity, and cell interactions. While size, shape, concentration, and exposure influence nanostructure toxicity, their effects vary across systems. Literature indicates morphology is not the primary toxicity factor. For gold nanorods, toxicity mainly depends on surface chemistry rather than aspect ratio (Wu et al., 2025).

Surface charge is vital in biology, as most membranes are negatively charged due to components like phospholipids and glycoproteins. Cationic nanoparticles bond strongly, induce internalization, damage membranes, and cause harmful enzyme buildup. Neutral or shielded surfaces can reduce nonspecific binding and increase circulation time. Studies show surface charge influences cellular uptake, membrane interactions, biodistribution, and toxicity (Alkilany et al., 2009; Khan et al., 2025). Surface functionalization allows decoupling near-infrared performance from biological reactions. Methods to reduce protein binding include polyethylene glycol, zwitterionic coatings, protein/saccharide shells, silica, and biomimetic membranes. Increasing hydrophilicity or steric stability can slow drug internalization and tumor accumulation, revealing a trade-off between biocompatibility and drug delivery. In UCNPs, silica coating thickness affects cytotoxicity, while PEG-like coatings influence circulation. Thus, optimizing particle size, shape, and surface charge should be viewed as interconnected design choices (Khan et al., 2025).

### 3.4 Degradation products and their toxicological implications

Structure–toxicity relationships focus on the long-term safety of NIR-responsive nanomaterials, especially their biological and photochemical fate after use. While toxicity assessments often examine the intact nanoparticle, nanomaterials can undergo hydrolysis, oxidation, enzymatic degradation, dissolution, photodegradation, or ligand exchange during circulation, cellular uptake, and NIR irradiation. These degradation products vary in size, solubility, charge, reactivity, permeability, and clearance. A nanoparticle with low acute toxicity may still produce toxic degradation products during prolonged exposure (Joshi et al., 2026). Proper degradation can turn persistent nanoparticles into smaller, water-soluble species that are easier to eliminate, potentially making them safer in the long term. This has led to the development of biodegradable and stimuli-degradable NIR photothermal agents engineered to disassemble in response to stimuli such as pH, enzymes, ROS, or NIR irradiation, particularly to address retention issues associated with nondegradable inorganic agents (Hossain et al., 2022). However, degradation does not automatically imply safety; the toxicological profile of the resulting products, including concentration, persistence, metabolism, cellular localization, and elimination, must be assessed independently. Metal-containing NIR-responsive systems exemplify the balance between degradation and toxicity (Khan et al., 2025). Under physiological conditions, CuS nanoparticles can degrade to soluble copper sulfate, thereby increasing copper excretion but increasing toxicity risks at high concentrations due to redox activity. The importance of studying the degradation process lies in analyzing the cytotoxicity of degradation products. The fate of UCNPs is governed by lattice dissolution and ion release, while surface coatings influence the rate of degradation and biological interactions (Ortiz de Solorzano et al., 2016).

These restrictions underscore the significance of safety-by-design principles in the material fabrication process, as well as biodegradability, shape and size control, biocompatible chemistry, low residual substance levels, predictable decomposition, and effective clearance of the material. NIR-absorbing substances must be optimized for effective photothermal conversion and production of reactive oxygen species as well as for photostability and dark toxicity. The manufacturing of nanoplatforms should help reduce toxic degradation products. The clinical translation must be supported by regulated NIR dosimetry, human-derived models, and pharmacokinetics, linked to the evaluation of degradation characteristics and long-term toxicity.

To adopt NIR-responsive nanomaterials in clinical practice, it is necessary to evaluate the material’s physicochemical properties, safety, manufacturing process quality, and irradiation parameters. Although various international organizations, such as the FDA, provide recommendations, there are still limitations with the models in common use. That is why further studies must implement a standardized evaluation system that includes biodistribution, degradation, clearance, immunotoxicity, and phototoxicity. Moreover, reproducible manufacturing and proper calibration of the NIR devices must be guaranteed, and the material must be consistent in all batches (He et al., 2016).

## 4 Disease microenvironments as safety modifiers

### 4.1 Reactive oxygen species (ROS)

It is necessary to state that oxidative stress is an essential safety modifier of the disease microenvironment because high ROS, such as H_2_O_2_, superoxide, hydroxyl radicals, and singlet oxygen, can selectively activate NIR-responsive nanomaterials (Malla et al., 2024). High levels of ROS can induce oxidative modification or fragmentation of ROS-sensitive chemical bonds, thereby affecting nanocarrier disassembly or the physical characteristics of the carrier surface, thereby increasing drug release or breakdown. This implies that ROS could act as a therapeutic activator and can also affect the stability, degradation, and pharmacological fate of the nanomaterial. Redox-responsive systems use oxidation-sensitive bonds to facilitate local degradation of nanocarriers and subsequent drug release (Chi et al., 2023). A liposomal delivery system responsive to ROS (Lipo/pB-DOX/ICG) was developed by Yi et al., in which ICG was used as the photosensitizer and ROS generator. At the same time, pB-DOX served as the ROS-responsive prodrug. Irradiation with 808 nm light triggered ICG-mediated ROS production, which could induce PDT and cleave the ROS-sensitive boronate group of pB-DOX, thereby regenerating doxorubicin for chemotherapy. The result obtained in this manner was 93.5% cell inhibition rate and 94.9% tumor growth inhibition rate *in vivo* (Yi et al., 2021) (Figure 3b). For instance, Zhou et al. formulated a lignin-based nanosystem that incorporates a doxorubicin prodrug linked via a thioketal bond cleavable under ROS conditions. Nanoparticles with a diameter of about 125.7 ± 3.1 nm induced ROS formation in 4T1 breast cancer cells but had minimal effect on L929 fibroblasts. Doxorubicin release was triggered by thioketal cleavage, resulting in efficient tumor inhibition and enhanced biosafety compared with free doxorubicin (Zhou Z. et al., 2024). A novel safety-focused approach by Tang et al. involved incorporating a BODIPY photosensitizer and ROS-cleavable thioketal linkages into biodegradable NIR-II fluorescent polymers, in which photogenerated ROS destroyed cancer cells and cleaved the thioketal linkages, thereby facilitating carrier decomposition and alleviating risks associated with cationic systems (Tang et al., 2024) (Figure 3. a). Moreover, in a separate approach, TC@PTP micelles were prepared by Zhou et al. using PEG5K-TK-PLGA5K (a porphyrin conjugate) and capsaicin. The drug release was mediated by ROS-activated cleavage of thioketal bonds, and capsaicin inhibited mitochondrial respiration and HIF-1 signaling, thereby reducing hypoxia and enhancing PDT at the tumor site (Shi et al., 2024). Conversely, Other thioketal-based systems have used ROS-induced bond cleavage to release immunomodulatory drugs or to enhance ROS generation, showing how microenvironment-responsive degradation can generate positive feedback in drug activity (Li Y. et al., 2025; Wang et al., 2023).

**FIGURE 3:**
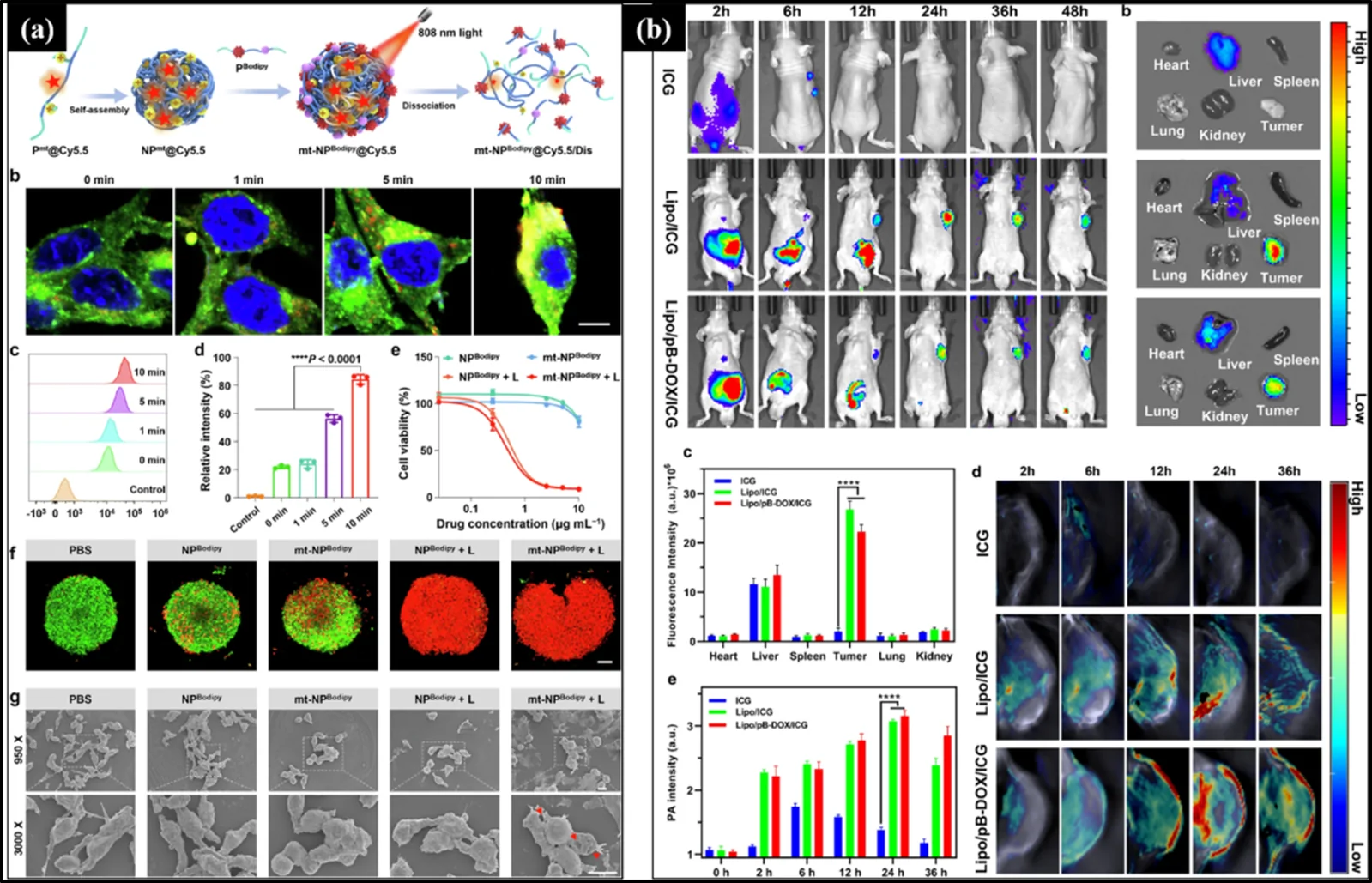
Representation of ROS-sensitive nanomaterial systems that demonstrate ROS-induced changes to the nanostructure, amplification of oxidative stress, responsive drug delivery, and biodistribution *in vivo*. **(a)** NIR-induced dissociation of mt-NP^Bodipy^ by means of ROS-induced thioketal cleavage, subsequent release of cationic polymers, and disruption of cancer cell membranes, adapted from (Tang et al., 2024) under the Creative Commons Attribution 4.0 International License https://creativecommons.org/licenses/by/4.0/, **(b)** *In vivo* fluorescence and photoacoustic biodistribution study of ROS-sensitive Lipo/pB-DOX/ICG nanoparticles versus free ICG and control liposomes showing tumor localization and near-infrared response of theranostic nanoparticles, adapted with permission from (Yi et al., 2021) under a Creative Commons Attribution 4.0 International License http://creativecommons.org/publicdomain/zero/1.0/.

Collectively, the above studies illustrate how ROS can regulate the disassembly of nanoparticles, drug release, disruption of membranes, oxygenation of the tumor, and immune response in a quantitative and mechanistic way; however, the same mechanisms used for ROS amplification and GSH depletion can lead to the oxidation of lipids, dysfunction of mitochondria, DNA/protein damage, and inflammation if the activation is not carried out within the target tissues. Therefore, safety testing needs to move beyond assessing cell viability and include ROS levels, the GSH/GSSG ratio, mitochondrial function, lipid peroxidation, inflammatory cytokines, degradation products, and tissue-specific oxidative injury.

ROS-activated NIR nanomaterial platforms, as well as their mechanism of activation, biological fate, therapeutic response, safety issues, and critical aspects of clinical translation, are provided in Table 1.

**TABLE 1:** NIR responsive ROS-activable nanomaterials: from microenvironment-activation to clinical translation.

Nanomaterials	NIR trigger	Microenvironment-responsive mechanism	Biological fate of nanomaterial	Key findings	Safety/toxicological profile	Clinical translation considerations	Ref.
Cluster bomb like nanomaterials	IR780-mediated NIR irradiation	Thioketal (TK) crosslinks respond to endogenous tumor ROS and NIR-generated ROS	∼135 nm particles accumulate in tumors; ROS/NIR-triggered size reduction enhances deep intratumoral penetration and cellular uptake	Enhanced PDT/PTT, IDO inhibition, and immunogenic cell death; improved primary-tumor suppression with abscopal/anti-metastatic effects	*In vivo* treatment showed no notable toxicity; however, the long-term fate of the PAMAM-derived fragments requires further investigation	Size transformation is attractive for improving tumor penetration, but reproducible degradation, fragment clearance, and long-term PAMAM safety require validation	(Zhang et al., 2021)
PDA nanoparticles	NIR activation of ZnPc photosensitizer	TK-linked PDA responds to intracellular ROS; ZnPc is initially quenched and becomes photodynamically active after ROS-mediated disassembly	PDA-Dox-Pc-QRH particles were ∼180 ± 9 nm by TEM; receptor-mediated uptake through EGFR targeting; intracellular ROS drives progressive disassembly	Dox loading ∼9 wt%; Pc loading ∼15 wt%; complete tumor ablation was reported in EGFR-positive tumor-bearing mice after systemic administration and irradiation	No notable toxicity was observed in the reported animal study; nevertheless, the authors explicitly identify the limited biodegradability of PDA as a translational concern	PDA-based materials can persist *in vivo*, making ROS-triggered degradation advantageous, but this requires confirmation of degradation products and clearance	(Dai et al., 2021)
Polymeric micelles	808 nm	ICG simultaneously generates ROS and heat; the TK linker is ROS-cleavable	Polymeric micelles showed tumor accumulation and improved photostability compared with free ICG; ROS-triggered disassembly promotes local GA release	97.9% tumor inhibition in a 4T1 breast cancer model; combined chemo-photothermal treatment showed strong antitumor activity	Reported preferable biocompatibility and negligible treatment-related side effects *in vivo*	Demonstrates self-sensitized ROS feedback; translation requires characterization of repeated-dose toxicity, degradation of TK-polymer conjugates, and ICG metabolism	(Yang et al., 2021)
Polymeric nanoparticles	808 nm	ROS-responsive oligomeric polymer undergoes ROS-triggered disassembly; ICG generates ROS following NIR irradiation	ROS-triggered disassembly facilitates intracellular payload release; the system was evaluated in a lung cancer model	Combined p53 mRNA expression and ICG-PDT produced an effective antitumor response and was reported as safe in the lung cancer model	The authors reported an effective and safe antitumor response; comprehensive long-term biodistribution and immunotoxicity remain important gaps	It is particularly promising for combining gene therapy and phototherapy, but mRNA stability, innate immune activation, repeated dosing, and scalable manufacturing remain barriers to translation	(Zhou et al., 2023b)
HSA-based nanoparticles	808 nm	IR780 produces ROS and heat; DTD contains a ROS-sensitive TK linkage	HSA-based nanoparticles facilitate systemic delivery and intracellular DOX release after NIR activation	Single NIR irradiation regulated PDT/PTT/chemotherapy and produced strong antitumor effects *in vitro* and *in vivo*	Nanoparticles showed good cellular/treatment compatibility in reported experiments; however, long-term HSA/IR780/DTD fate requires further study	Single-wavelength activation simplifies treatment, but optimizing laser dose and controlling ROS/thermal exposure are important for clinical translation	(Chen et al., 2024b)

### 4.2 Acidosis

Acidosis is a significant pathology-induced microenvironmental safety modulator, as the slightly acidic nature of tumor and inflammatory tissue environments can affect nanoparticle surface charge, stability, degradation, aggregation, cellular uptake, and drug release. Therefore, multiple NIR-responsive systems remain stable in circulation while undergoing alteration in the pathologic environment. For instance, Zhuang et al. designed an oxygen-abundant, pH/NIR dual-responsive nanocarrier, in which a chitosan shell facilitated DOX release via an acidic pH response, while 808 nm irradiation induced photothermal heating, thereby enhancing drug release. The perfluorocarbon supplied oxygen, inhibited HIF-1α protein expression in tumors, and enhanced PDT efficacy under hypoxia, while the dual-triggering system aimed to minimize nonspecific toxicity from premature drug release (Zhu et al., 2021). Another important example is the pH-responsive NIR-II PTDG nanoplatform developed by Zhang et al., in which cleavage of the acid-labile ester linkages at pH 6.8 released the vasculature-disrupting drug DMXAA, thereby forming photothermal PT-3 particles. In addition, the nanoparticles displayed an NIR-II brightness value of 119.61 M^-1^ cm^-1^, a circulation half-life of 235.6 min, and a photothermal conversion efficiency of 40.5%; more importantly, a complete inhibition of the tumor was achieved when the combination of anti-vascular and photothermal therapy was applied, with the particles remaining inside the tumors for over 2 weeks. In another report by Fan et al., a pH-dependent mechanism of activation of Fe_3_O_4_/CaCO_3_-CSL/ICG nanoparticles was established, in which the dissolution of CaCO_3_ at acidic pH led to Ca^2+^ loading, creating intracellular calcium overload and mitochondrial damage, and further amplifying these effects through the NIR photothermal and photodynamic properties of ICG-loaded nanoparticles. Especially relevant from a safety-by-design perspective, the pH-responsive tBT@PANI-MnO_2_ material demonstrated that acidity could modulate its photothermal properties. At pH 6.5, the photothermal conversion efficiency of the material was about 4.67 times higher compared to pH 7.4, while the pyroelectric potential was increased by about 1.63 times (Fan et al., 2025). Soman et al. demonstrated a DOX- and PDA-containing, pH- and NIR-sensitive, transferrin-functionalized black phosphorus nanodelivery platform that exhibited negligible hemolytic and CAM toxicity but increased ROS production, apoptosis, and necrosis, and anti-tumor effects under NIR stimulation. Similarly, an acidity- and size-adaptability-responsive nanoparticle system incorporating hyaluronidase, acidic pH, and NIR stimuli was employed for sequential structural modification, deep tumor penetration, and dual DOX chemotherapy with IR780-mediated photothermal and photodynamic therapy (Fan et al., 2025) (Figure 4b). Jia et al. described the use of Fe_3_O_4_-graphene oxide nanocomposites modified with hyaluronic acid and folic acid as dual pH/NIR-sensitive systems for chemotherapy and photothermal therapy in combination (Jia et al., 2026) (Figure 4a), Li et al. created a novel acid/NIR AND-logic gate for the selective and controlled release of nitric oxide under acidic and NIR conditions.

**FIGURE 4:**
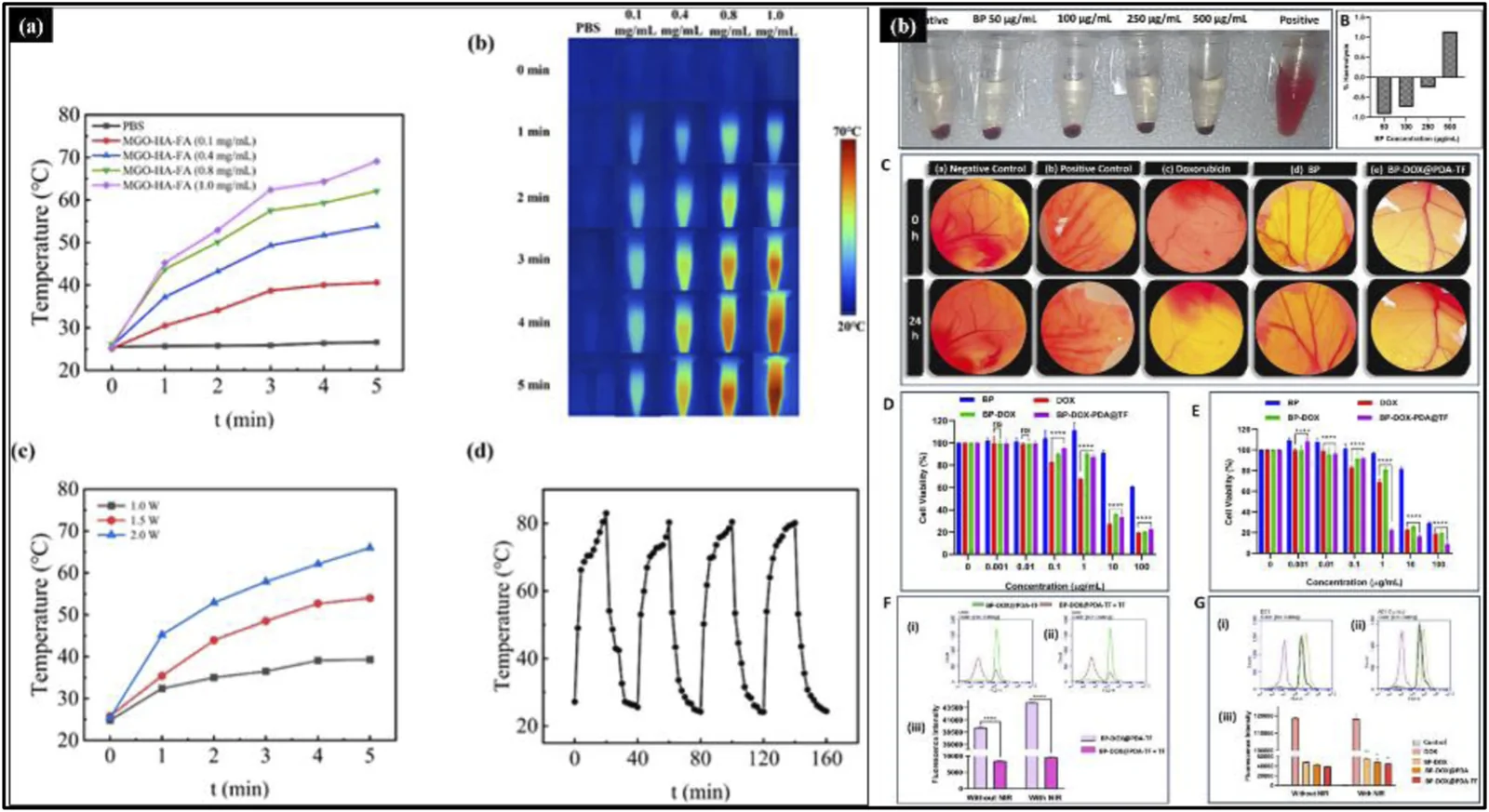
pH-activatable and NIR-activatable nanocarrier systems exemplifying pH-responsive drug release, photothermal response, biodistribution, and therapy safety. **(a)** NIR-Induced Photothermal Effect of DOX/MGO-HA-FA Nanoparticles, including effects of concentration and exposure to light, infrared thermal imaging, intensity-dependent effect, and photothermal stability upon multiple exposures to light, adapted with permission from, under Creative Commons CC BY 3.0, https://creativecommons.org/licenses/by/3.0/. **(b)** Evaluation of hemolytic effect, CAM Assay, cell viability, and flow cytometric analysis of BP-DOX@PDA-TF and its derivatives, establishing the biocompatibility and NIR-induced therapy using pH/NIR-responsive BP nanoplatform, adapted with permission from (Soman et al., 2025), under the terms of the Creative Commons CC-BY license https://creativecommons.org/licenses/by/4.0/, *© 2025 The Authors. Published by Elsevier* *B.V.*

Studies show disease-induced acidity influences more than drug release, affecting nanoparticle protonation, charge conversion, disassembly, size change, metal ion liberation, ROS generation, photothermal conversion, imaging activation, and apoptosis pathways. This raises toxicological concerns as acidic conditions occur in tumors, inflamed, ischemic, and infected tissues. Excessive acid-induced degradation leads to uncontrolled drug release, exposure to metal ions, ROS production, or altered nanoparticles. Transitioning from pH-sensitive to dual pH/NIR and AND-activated systems offers safety-by-design, with endogenous acidity ensuring selectivity and NIR providing external control. Future research should assess nanoparticle stability, premature release, degradation, biocompatibility, inflammation, biodistribution, clearance, and long-term tissue accumulation, considering factors like hypoxia, acidosis, and microbial stimuli that modulate ROS and nanomaterial responses.

Selective pH-sensitive NIR nanoplatforms, along with their mechanism, fate within the body, efficacy, toxicity, and translational issues, are presented in Table 2.

**TABLE 2:** pH-responsive NIR activable nanomaterials: pH-responsiveness to their clinical translations.

Nanomaterial	NIR trigger	Miceroenvironment-responsive mechanism	Biological fate of nanomaterial	Key quantitative findings	Safety/toxicological profile	Clinical translation considerations	Ref.
Gold nanoclusters	NIR laser	Protonation of the PABI component under acidic conditions increases pH responsiveness and facilitates DOX release	Dox/GNC-loaded PEG-PABI nanoparticles were approximately 120 nm and showed pH-sensitive behavior	Repeated NIR exposures maintained photothermal performance and produced a strong synergistic chemo-photothermal effect	The system showed favorable therapeutic compatibility in the reported models	Repeated NIR treatment is attractive but requires clinically realistic irradiation schedules and adequate tissue penetration	(Husni et al., 2023)
Lipidic nanoparticles	NIR-II	Protonation under acidic tumor conditions switches the optical state and promotes activation	Tumor acidity enables preferential activation in the tumor microenvironment	pKa values around the tumor-acidic range allow discrimination between physiological and tumor pH	pH activation can reduce systemic phototoxicity, but prolonged retention must be evaluated	pKa optimization is crucial because tumor acidity is heterogeneous and differs between tumor types	(Chai et al., 2024)
Polymeric nanoparticles	NIR irradiation	Acidic pH facilitates DOX release from the pH-responsive nanostructure; BP/PDA components provide NIR photothermal activity	Transferrin targeting promotes tumor-cell uptake; pH and NIR jointly regulate therapeutic activation	NIR irradiation significantly increased cancer-cell killing and tumor suppression relative to nonirradiated treatment	Low hemolysis and favorable CAM compatibility were reported; long-term BP degradation products require evaluation	Biodegradable BP is attractive, but its degradation products, phosphorus metabolism, and chronic biodistribution must be established	(Soman et al., 2025)
Magnetic nanoparticles	NIR irradiation	Acidic conditions dissolve CaCO_3_ and alter the nanoparticle microenvironment; ICG generates ROS	Acid-sensitive components promote intracellular/tumor-localized activation; ICG enables simultaneous PDT/PTT	Combined Ca^2+^ overload and ICG-mediated PTT/PDT enhanced tumor-cell killing	Calcium-containing components may have favorable degradability, but Fe_3_O_4_ persistence and long-term organ accumulation need evaluation	Acidosis could directly cause toxicity through changes in intracellular ion homeostasis and nanoparticle-induced cellular effects, rather than merely serving as a trigger for drug release	(Fan et al., 2025)
Polymeric nanovesicles	NIR-II	Protonation of P4VP under acidic conditions destabilizes the nanovesicle	Nanovesicles remain stable under physiological conditions but disassemble in acidic tumors	pH-triggered activation enabled NIR-II imaging and enhanced radiotherapy	Reduced premature activation is advantageous; however, Ag_2_S/Au-containing components raise long-term clearance concerns	A strong example of acid-triggered structural transformation → activatable imaging, but persistent inorganic components remain a translational challenge	(Zhao et al., 2025)
Inorganic nanoparticles	NIR irradiation	Acidic H_2_O_2_-rich TME promotes MnCO_3_ dissolution and therapeutic activation	Acid-sensitive MnCO_3_ facilitates tumor-localized degradation and release of DOX/IR820	Combined chemotherapy/PTT/PDT enhanced tumor inhibition relative to individual treatments	BSA provides biocompatibility; systemic manganese exposure and degradation/clearance require further investigation	Biodegradable carbonate-based components offer a potential advantage over persistent metal nanostructures	(Li et al., 2025b)
Inorganic nanoparticles	NIR irradiation	Acidic pH promotes pH-sensitive structural/chemical changes and drug release	HA/FA functionalization promotes tumor-cell interaction and tumor accumulation	Dual pH/NIR activation produced enhanced chemo-photothermal antitumor activity	GO and Fe_3_O_4_ introduce potential long-term accumulation concerns despite active targeting	Multifunctional targeting + acidity + NIR is attractive, but increasing component complexity may complicate regulatory characterization	(Jia et al., 2026)

### 4.3 Hypoxia

A key hallmark of many solid tumors is the presence of hypoxia, which presents a significant biological and toxicological hurdle for the use of NIR-responsive theranostic platforms due to their dependence on oxygen for normal functioning and due to the progression of tumor growth, resistance, angiogenesis, and metastasis. Instead of viewing hypoxia solely as a barrier, modern NIR-responsive nanoplatforms leverage it as a therapeutic tool. The interactions between hypoxia and nanomaterials are two-sided: the hypoxic microenvironment itself can influence the oxygen availability and redox status of cells, thereby affecting ROS formation, photodynamic efficiency, drug degradation, and drug activation. The other way around, nanomaterials can also influence local oxygen availability by consuming, producing, or redistributing oxygen. Dutta et al. designed ICG-loaded hypoxia-sensitive polyprodrug micelles such that NIR radiation triggered ICG-mediated ROS production, thereby consuming oxygen within the tumor microenvironment and leading to hypoxic stress and accelerated release of hypoxia-sensitive camptothecin. It is noteworthy that this research is particularly relevant, as it demonstrates that ICG-mediated oxygen depletion can itself serve as a drug-delivery signal (Dutta et al., 2021). Consequently, some of the platforms have adopted the use of PFCs, ZnO_2_, Pt-based materials, or catalytic processes to raise the levels of oxygen in the environment and activate PDT (Xavierselvan et al., 2022; Wu et al., 2022; Jiang et al., 2022; Lu et al., 2023). Liu et al. developed an ICG-based Ce^4+^ nanosystem that stabilized ICG, promoted ROS generation, decomposed H_2_O_2_ to address tumor hypoxia, and reduced GSH. This system overcame ICG drawbacks such as aggregation, instability, and inefficient ROS production, thereby increasing tumor cell damage. However, Ce^4+^ catalysis could cause nonspecific ROS and toxicity in normal tissues. Alternatively, ICG–photosynthetic hybrids using cyanobacteria enable oxygen production via photosynthesis, overcoming tumor hypoxia and leveraging ICG’s photodynamic effects for NIR therapy.

In summary, these investigations clearly show that hypoxia can significantly influence the pharmacology of NIR-activatable materials, which can be regulated by supplying oxygen via perfluorocarbon administration, oxygen production via the catalytic action of ZnO_2_, Pt, catalase, or Ce^4+^, biological oxygen generation, oxygen consumption inhibition, and oxygen depletion for activation of hypoxia-sensitive prodrug. Still, these mechanisms of action must be considered in light of their toxicity. Overproduction of oxygen, followed by ICG-induced ROS formation, may increase oxidative stress in neighboring healthy tissues, while metal-containing oxygen generators may exacerbate metal-ion release, accumulation in organs, inflammation, and long-term excretion. At the same time, oxygen depletion may activate HIF pathways and angiogenesis if hypoxia-sensitive therapeutics are not fully activated. For this reason, studies on ICG-based hypoxia-responsive nanomaterials should focus not only on antitumor activity but also on intratumoral oxygen concentration, HIF-1α/HIF-2α expression, ROS/RNS generation, GSH consumption, mitochondrial respiration, inflammation, biodistribution, degradation, metal ion release, and long-term tissue localization. Since hypoxia often coexists with high ROS, extracellular acidosis, and inflammatory factors, the impact of hypoxia on nanomaterials must be understood in the broader context of the microenvironment rather than as a lone variable.

A summary of representative hypoxia-responsive NIR nanoplatforms, along with their mechanisms, biological fates, therapeutic effects, safety profiles, and clinical translations, is provided in Table 3 below.

**TABLE 3:** NIR responsive hypoxia-activable nanomaterials: From their hypoxia responsiveness to their clinical translation.

Nanomaterial	NIR trigger	Microenvironment responsive mechanism	Biological fate of nanomaterial	Key quantitative findings	Safety/toxicological profile	Clinical translation considerations	Ref.
Polymeric nanoparticles	NIR activation of ICG	Oxygen-loaded PFTBA supplies O_2_ to overcome hypoxia; ICG generates ROS after NIR irradiation	Cancer-cell-membrane-coated HSA–ICG–PFTBA nanoparticles provide homologous tumor targeting and local oxygen delivery	Increased tumor oxygenation enhanced PDT and tumor suppression in 4T1 xenografts;^18^F-FMISO PET/CT was used to monitor tumor hypoxia	ICG, HSA, and PFTBA were highlighted as clinically relevant components; biomimetic membrane safety and long-term fate still require evaluation	The use of clinically familiar ICG/HSA/PFC components is attractive, but cancer-cell membranes pose challenges in manufacturing, immunogenicity, and batch consistency	(Fang et al., 2021)
​	NIR activation of ICG	ICG produces ROS while hypoxia activates tirapazamine (TPZ)	PDA@ICG-TPZ nanoparticles were camouflaged with cRGD-modified tumor-cell membranes for tumor targeting	Combined PDT/PTT/TPZ treatment enhanced tumor killing under hypoxic conditions	Biomimetic membrane coating can reduce immune recognition; the long-term fate of PDA and membrane components requires investigation	Intentional use of hypoxia amplification to activate a hypoxia-sensitive chemotherapeutic rather than attempting to eliminate hypoxia	(Guo et al., 2022)
Silica nanoparticles	NIR irradiation	ICG acts as a photothermal converter that ruptures the silica shell and releases stored O_2_	PLGA drug-loaded core + ultrathin silica shell + Gd-DTPA; RGD/uPA targeting promotes tumor accumulation; MRI enables biodistribution monitoring	NIR-triggered O_2_ release relieved hypoxia, enhanced chemotherapy/PTT, and suppressed metastasis/EMT; no toxic side effects were reported	The study reported no significant toxic side effects and used MRI to monitor distribution	Strong theranostic design, but Gd-containing components and silica-shell degradation require long-term clearance assessment	(Zhang et al., 2022)
Metal Organic Frameworks	808 nm NIR	PFH provides oxygen modulation; ICG-mediated PDT is supported by oxygen availability	MOF-based platform combines ICG, perfluorohexane, and DNA-Dox; targeting components improve tumor localization	A single 808 nm laser enabled synergistic chemotherapy/PDT/PTT with hypoxia modulation	Targeting was reported to reduce off-target effects; MOF/PFH long-term fate requires investigation	Integration of imaging, oxygen modulation, and therapy on a single platform is promising, but MOF degradation and reproducibility remain key barriers	(Lin et al., 2024)
Inorganic nanozymes	NIR irradiation of ICG/IR820	CeO_2_, Fe_3_O_4_, and MnO_2_ function as oxygen-generating nanozymes to improve PDT under hypoxia	Nanozymes were evaluated in FaDu oral-carcinoma spheroids as a 3D hypoxic tumor model	Optimized oxygen-generating nanozyme combinations substantially increased phototherapy efficacy; oral carcinoma spheroids completely regressed under optimized irradiation conditions	Use of inorganic nanozymes raises questions regarding persistence, ion release, and long-term tissue accumulation	The use of 3D tumor spheroids is valuable for safety/efficacy prediction because oxygen gradients more closely mimic those of solid tumors than 2D cultures	(Kothari et al., 2024)
Inorganic nanoparticles	NIR/ICG-mediated phototherapy	MnO_2_ converts endogenous H_2_O_2_ into O_2_; metformin decreases cellular oxygen consumption	H-MnO_2_ nanoparticles decompose in response to GSH/acidic TME and release Mn^2+^, Fe^2+^, ICG, and metformin; FA promotes tumor-cell uptake	Dual O_2_-generation/O_2_-economization strategy enhanced CDT/SDT and reduced hypoxia	Multifunctional metal-containing system showed satisfactory therapeutic efficacy; Mn/Fe exposure and degradation products require chronic safety evaluation	Simultaneous oxygen generation and oxygen consumption suppression, which may be more effective than oxygen supplementation alone	(Zhang et al., 2025b)

### 4.4 Microbial microenvironment

The microbial environment, including EPS, enzymes, pH, redox conditions, and pathogen interactions, influences penetration, aggregation, biodegradation, stability, activation, and payload delivery, affecting antimicrobial efficacy and cytotoxicity. Chen et al. created berberine hydrochloride/ICG nanoparticles via electrostatic and π–π interactions, achieving 36% photothermal efficiency. Under NIR, 67.73% of berberine was released, with dual photothermal and antibacterial effects that remove *Staphylococcus aureus* and aid wound healing, illustrating how NIR-induced changes can enhance drug delivery. Zhou et al. produced Au@mSiO_2_-Arg/ICG nanoparticles that combine ROS generation, photothermal effects, and nitric oxide release via L-arginine. With 808 nm irradiation at 0.8 W/cm^2^, they eliminated 99.98% of biofilms, as NO suppressed heat-shock protein 70 and EPS, increasing bacterial susceptibility to heat and improving penetration. This study highlights microbial environments as safety modifiers, showing that bacterial responses can limit photothermal effects and that targeting these responses allows bacteria to be killed at lower temperatures, thereby reducing tissue damage (Figure 5b). Qi et al. designed Prussian blue-functionalized ICG-loaded mesoporous silica nanoparticles against MRSA by combining NIR-triggered PTT/PDT with chemodynamic therapy via H_2_O_2_. This therapy leveraged the oxidative nature of infection to generate additional hydroxyl radicals, while ICG served as a source of photothermal and photodynamic therapy (Qi et al., 2024). Various systems have used the sensitivity of microorganisms to ROS, quorum-sensing pathways, bacterial surface interactions, and biofilm structural features to boost the antimicrobial activity of NIR light. Such systems would be capable of boosting bacterial targeting, disrupting biofilm-associated protection, and enhancing ROS-mediated damage to microorganisms (Solarte et al., 2022; Xu P.-Y. et al., 2022; Xu Y. et al., 2022).

**FIGURE 5:**
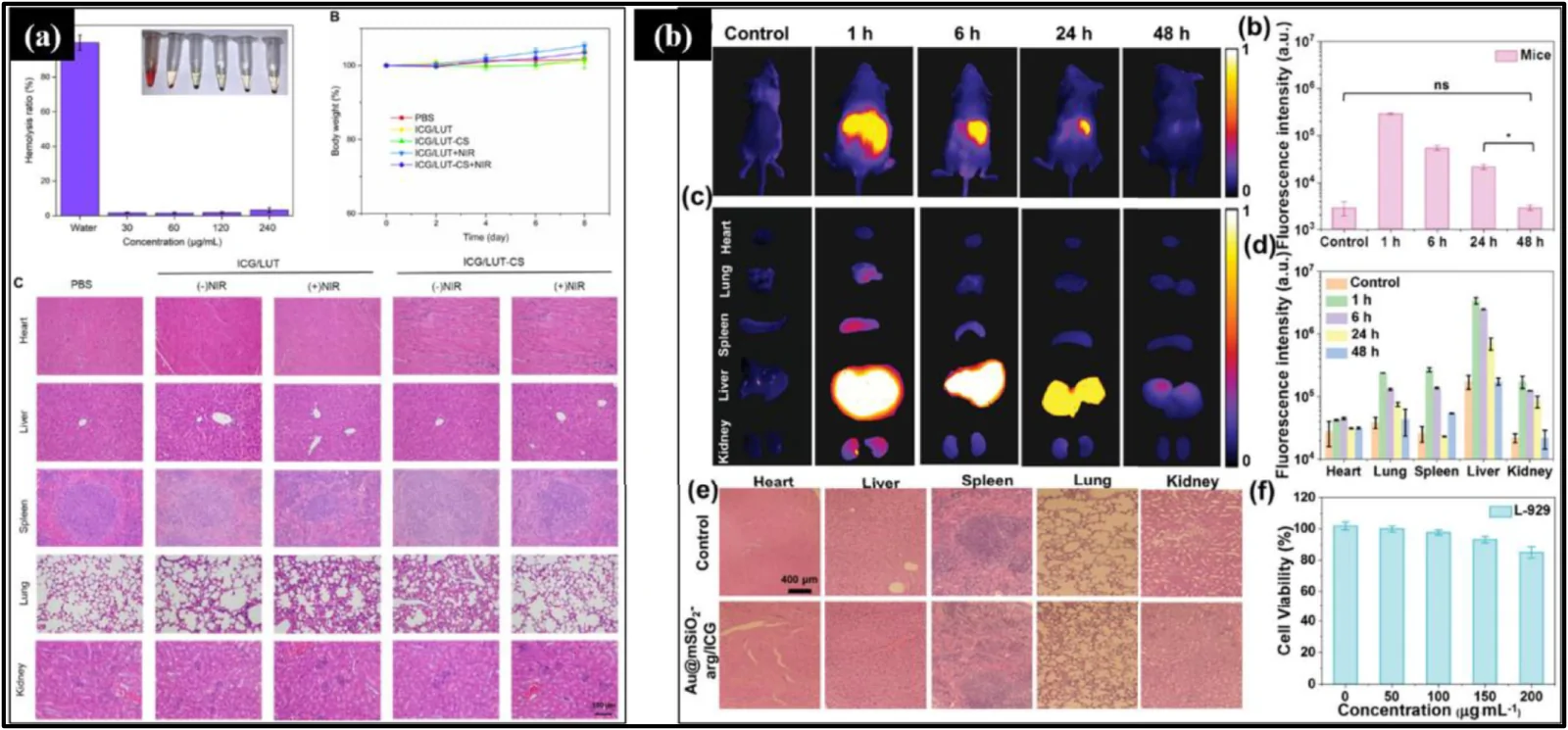
*In vivo* biological interaction study, antimicrobial effect, and biosafety test on NIR-triggered nanosystems for bacterial infection therapy. **(a)** Hemolytic analysis of ICG/LUT-CS at various concentrations, change in weight of mice during the treatment period, and photomicrographs of major organs stained with H&E, showing good systemic biocompatibility of ICG/LUT-CS nanocomposite, adapted from (Xu P.-Y. et al., 2022) under Creative Commons CC BY license https://creativecommons.org/licenses/. **(b)** *In vivo* fluorescence imaging, *ex vivo* organ distribution, and histological study on major organs, as well as the cytocompatibility study of Au@mSiO_2_-Arg/ICG, which shows the biodistribution and initial safety of Au@mSiO_2_-Arg/ICG after NIR-light-triggered NO-releasing mild-temperature photothermal/photodynamic therapy, adapted with permission from (Zhou B. et al., 2024), *© 2024 Wiley‐VCH GmbH*.

These studies show that the microbiological environment influences NIR-responsive ICG systems through biofilm formation, the EPS barrier, bacterial stress responses, QS, enzymatic activity, oxidative conditions, and pathogen-host interactions. ICG phototherapy also affects the microbiological environment through bacterial membrane disruption, EPS degradation, ROS production, localized heating, and stress-response modulation. However, these interactions raise safety concerns, as ROS and photothermal effects can damage nearby healthy tissue, including keratinocytes, fibroblasts, and mucosa, and ROS may also adversely affect beneficial microbiomes. Therefore, infection-specific, enzyme-based, and bacteria-targeted therapies that allow therapeutic activation and targeted delivery of nanomaterials to infection sites are crucial safety strategies, as they restrict treatment and nanoparticle buildup to affected areas.

A summary of representative microbial microenvironment-responsive NIR nanoplatforms, along with their mechanisms, biological fates, therapeutic effects, safety profiles, and clinical translations, is provided in Table 4 below.

**TABLE 4:** NIR and microbial microenvironment responsive nanomaterials and their mechanism, biological fate, key findings, safety profile, and clinical translation considerations.

Nanomaterial	NIR trigger	Microenvironment-responsive mechanism	Biological fate of nanomaterial	Key quantitative findings	Safety/toxicological profile	Clinical translation considerations	Ref.
Polymeric nanoparticles	NIR laser; photothermal activation of ICG	Acidic/infection-associated environment promotes drug release; luteolin inhibits bacterial quorum sensing, and ICG generates ROS/heat	Chitosan-coated ICG/LUT nanoparticles interact with infected wound tissue and bacterial biofilms; LUT interferes with quorum-sensing signaling and biofilm formation	Enhanced bacterial killing an,d biofilm elimination; synergistic chemotherapy/PTT promoted infected-wound healing	Chitosan and luteolin offer favorable biocompatibility; systemic toxicity and long-term retention require further evaluation	Disruption of quorum-sensing signaling is yet another approach for interfering with the processes of bacteria that form biofilms	(Xu et al., 2022a)
Organic nanoparticles	808 nm, NIR irradiation	ICG generates ^1^O_2_ and heat; bacitracin provides bacteria-targeting/antibacterial activity	BSA@ICG nanoparticles are surface functionalized with positively charged bacitracin, increasing interaction with negatively charged bacterial surfaces	Enhanced photodynamic/photothermal antibacterial activity and biofilm removal compared with BSA@ICG; effective against bacterial pathogens	Reported good biocompatibility; BSA-based carrier reduces some formulation concerns, although repeated-dose immunogenicity requires evaluation	Bacterial-surface targeting and NIR phototherapy can improve selectivity and potentially reduce the NIR dose required for antimicrobial activity	(Xu et al., 2022b)
Zirconia surface	810 nm diode laser	ICG acts as the NIR photothermal/photodynamic agent	ICG is localized at the zirconia-associated biofilm; no nanoparticle carrier is required	Significant reduction of *S. gordonii* was observed in PDT/PTT groups; the highest dead bacteria count occurred in PDT/PTT and chlorhexidine groups	Local application minimizes systemic exposure; study was performed *in vitro*, so systemic toxicity/clearance was not addressed	Demonstrates that NIR/ICG can target surface-associated oral biofilms, relevant to dental implants and biomaterial-associated infection	(Shim et al., 2022)
Self-assembling nanoparticles	NIR irradiation	ICG produces photothermal/photodynamic effects; berberine provides direct antibacterial activity	Carrier-free BH/ICG nanoparticles self-assemble through electrostatic/π–π interactions and release BH under NIR	Photothermal conversion efficiency reached ∼36%; NIR-triggered BI NPs showed enhanced antibacterial activity and promoted *S. aureus*-infected wound healing	Reported good biocompatibility; carrier-free formulation reduces excipient burden, but ICG/BH pharmacokinetics require further study	Carrier-free assembly can simplify translation by reducing carrier-related toxicity and manufacturing complexity	(Huang et al., 2023)
Silica nanoparticles	NIR irradiation	H_2_O_2_-responsive Prussian blue provides chemodynamic activity; ICG provides PDT/PTT	Mesoporous silica nanoparticles provide a reservoir for ICG/PB; bacterial infection-associated H_2_O_2_ contributes to activation	Strong antibacterial activity against MRSA and biofilms; synergistic triple-mode therapy	Reported satisfactory biosafety; long-term fate of silica/PB and iron-associated degradation products remains to be established	An important example of using an infection-associated biochemical cue (H_2_O_2_) together with external NIR to improve selectivity	(Qi et al., 2024)
Microneedles	NIR irradiation	ICG generates ROS and heat; HA/ZIF architecture improves interaction with infected tissue/biofilm	ICG@ZIF@HA is incorporated into a microneedle patch, providing local delivery directly to the infected tissue	Efficient biofilm elimination was demonstrated; the microneedle platform improved localized phototherapy	Localized administration reduces systemic exposure; ZIF degradation and repeated application require safety assessment	Particularly promising for clinical translation because microneedle-mediated local delivery largely avoids the systemic biodistribution problem associated with injectable nanoparticles	(Zheng et al., 2025)

### 4.5 Crosstalk between microenvironmental factors and safety implications

Conditions like hypoxia, acidosis, ROS, and redox imbalance are usually found simultaneously in tumors and affect various processes related to nanoparticles (Zhang, 2025). While PFC/ICG exploits hypoxia in tumors and pH-sensitive systems target acidity in tumors, high levels of ROS or hypoxia may cause an increase in oxidative stress (Ruan, 2025). Acidic and oxidative environments may work together, thus hastening the process of degradation of nanomaterials, as well as the release of drugs (He et al., 2025). The same principle is at play within the microenvironment of microbes, wherein the biofilms contain gradients of pH, oxygen, and EPS, which influence the penetration of nanoparticles, whereas ROS generated by NIR light damages the bacterial cell membrane (Ali et al., 2024). Thus, safety evaluation should assess combined microenvironmental stimuli and their effects on nanoparticle stability, degradation, ROS generation, drug release, surface charge, and cellular uptake. 3D spheroids, organoids, biofilm, infected-tissue, and relevant *in vivo* models can better determine whether NIR activation remains localized to diseased tissues (Park et al., 2021).

## 5 Biological degradation and toxicological effects

The biological breakdown, intracellular metabolism, distribution, localization, and removal from the body of ICG-responsive nanomaterials are crucial for their safety and translational potential, especially when the nanoparticles alter the fast pharmacokinetics of the free dye. According to Ding et al., the biological environment is capable of significantly affecting the structural form and the localization of ICG nanoparticles, with the J-aggregated ICG nanoparticles being gradually dissolved into monomeric ICG in the tumors, such that tumor-specific ICG fluorescence attained its peak level around 48 h post administration, but not the J-aggregated ICG, which localized in the reticuloendothelial system organs. Similarly, Kwon et al. have shown that the ICG dimerization due to nanostructure design can significantly enhance the *in vivo* stability of ICG assemblies; the stable Nano-dICG formulation was able to provide a photoacoustic signal in tumor, liver, and spleen tissues 24 h after administration, while the unstable ICG formulation had no photoacoustic properties after exposure to the biological environment. Cell internalization is another critical factor influencing cytotoxicity, as the intracellular accumulation of ICG and carrier material in endosomes, lysosomes, mitochondria, and other organelles can extend the photothermal and ROS effects well beyond the irradiation time. In particular, this becomes significant for nanoparticles designed to enhance cell uptake via receptor-mediated targeting; in such cases, increased uptake by tumor cells may be paralleled by increased uptake by macrophages and other components of the mononuclear phagocytic system (Kwon et al., 2023). Hyaluronic acid modifications to ICG nanoparticles by Wang et al. have been found to alter their biodistribution and metabolism *in vivo*, as determined by fluorescence assays, with results indicating liver accumulation followed by the kidneys at 6, 24, and 48 h (Gao et al., 2024). Al-Jamal et al., in more recent times, examined biodegradable lipid bilayer-assisted ICG J-aggregates and found that, although the preparation enhanced the stability of ICG aggregates, it was still prone to biological degradation; notably, this study included both *in vitro* toxicity tests and *in vivo* clearance studies, demonstrating that the biodegradable lipid part of such preparations might provide a solution to the problem of persistence of ICG inorganic nanocarriers. Indeed, biodistribution studies have shown that nanoparticle formulation may alter the fate of ICG compared to free dye. Since free ICG is rapidly taken up by hepatocytes and eliminated via the hepatobiliary pathway, the nanoparticle formulation may enhance its circulation time and distribution in the liver, spleen, kidneys, lungs, and other organs. For instance, organ-level quantification of ICG nanocarriers has shown considerable accumulation in the liver, with distribution in the spleen, lungs, kidneys, and intestine, indicating the significance of the reticuloendothelial system in nanoparticle accumulation and subsequent clearance (Al-Jamal et al., 2025).

The development of the protein corona greatly impacts the biological nature, biodistribution, target specificity, and toxicity of near-infrared (NIR)- responsive nanoparticles. The nanoparticles adsorb proteins such as albumin, immunoglobulins, apolipoproteins, and complement proteins upon contact with biological fluids, depending on the physicochemical characteristics of the nanoparticles and their biological environment (Wheeler et al., 2021; Monopoli et al., 2012). Depending on the nature of the corona composition, it can either enhance receptor-mediated entry or facilitate immune recognition and clearance of the nanoparticles, thereby impacting their circulation time, accumulation within tumors, and targeting to specific organs, including the liver and spleen. The modifications can further affect the uptake and localization of NIR-responsive photothermal or photosensitizing agents, thereby altering their efficacy and the potential to induce unwanted effects within tissues (Wang J. et al., 2024; Mahmoudi et al., 2023). Recent research by Zhang et al. used 7-nm PEGylated carbon nanodots with an ICG coating, showing that the serum protein corona affected interactions with cells and reduced macrophage uptake. The nanoparticles had an IC_50_ > 500 μg mL^-1^ across several cancer-cell models, accumulated in a time-dependent manner in tumor tissue, liver, and kidneys, and showed no significant hepatic or renal toxicity or inflammation during the observation period. This work highlights the protein corona as a key factor influencing cellular uptake: serum protein adsorption impacts nanoparticle surface properties, macrophage recognition, intracellular trafficking, and biodistribution. Extended accumulation poses greater risks for non-biodegradable inorganic cores compared to free ICG or biodegradable systems. The persistence of ICG aggregates or ICG-functionalized nanoparticles in liver, spleen, or tumor tissue, even after free dye has cleared, suggests that carrier persistence may be the main safety concern (Cillari et al., 2026). As shown by Ding et al., the breakdown in the integrity of the J-aggregates was related to their transformation into monomeric ICG, which indicates that the eventual breakdown in structure can contribute to the process of ICG removal; however, the resistance of the carrier matrix to degradation may be an obstacle for clearance even after the release of ICG. Thus, the removal process will greatly depend on the size of the nanoparticles, surface charge, composition, state of aggregation, and biodegradability (Farrakhova et al., 2022). The significance of the biodegradability of lipid constructs in the clearance of ICG aggregate was illustrated by Al-Jamal et al. (2025). In contrast, the hepatic and renal metabolism of HA-functionalized ICG nanoparticles was observed by Wang et al. (Zhang et al., 2024). It is important to distinguish between dye clearance and carrier clearance, as NIR fluorescence clearance does not reflect total nanomaterial clearance due to deaggregation, degradation, photobleaching, or ICG metabolism, even when the carrier remains in tissues. This issue is more pronounced in the NIR-II system, which allows tracking in deep tissues due to reduced quenching. For thorough toxicological analysis, techniques like whole-body fluorescence, photoacoustic imaging, tissue quantification, and histopathological, biochemical, immunological, and elemental analyses are essential. Focus should include cellular internalization, intracellular localization, macrophage uptake, deposition in liver and spleen, renal involvement, carrier degradation, dye release, toxicity of degradation products, hepatobiliary vs. renal clearance, and persistence beyond the therapeutic window. Overall, safety of NIR theranostics cannot be assumed from the free ICG’s clinical profile, as loading into nanoparticles may alter its biological behavior. Degradability and clearance are key safety considerations, especially for agents meant for repeated use.

## 6 Phototoxicity and immunotoxicity

Phototoxicity in NIR theranostics involves understanding both dark and photoinduced toxicity, particularly with ICG platforms that generate ROS, which can cause oxidative damage and cell death. Yang et al. showed that excipient-free ICG nanoparticles enhance ROS generation, thereby increasing PDT efficacy but also raising oxidative damage risks. ROS can cause mitochondrial and DNA damage, while heating may cause protein denaturation and necrosis. Effects depend on ICG concentration, localization, light parameters, oxygen, and temperature. Photoactivation can also trigger immunogenic cell death, marked by calreticulin, ATP, and HMGB1 release (Yang et al., 2024). Zhang et al. showed that ICG-encapsulated nanoparticles induced ICD upon NIR irradiation and promoted antigen presentation, demonstrating how phototoxicity can be converted into a systemic antitumor immune response (Wang C. et al., 2024). Likewise, Sun et al. (2025) formulated ICG J-aggregates along with a STING agonist to combine photothermal ablation of tumors with innate immune response. But activation of the immune response must be clearly distinguished from immunotoxicity, as excessive ROS production, nanoparticle loading, and excessive activation of macrophages and dendritic cells can lead to the production of cytokines, including IL-1β, IL-6, and TNF-α, and initiate unnecessary systemic inflammation (He et al., 2025). Sun et al. (2026) further showed that a combination of photothermal treatment based on ICG and PKM2 gene knockdown suppressed HSP70-induced thermotolerance and enhanced the immune response (Sun et al., 2026). Therefore, the evaluation of safety for NIR-triggered systems should consider quantifying dark toxicity and photoinduced toxicity, distinguishing ROS-related injury from thermal effects, and distinguishing physiological immune response from pathological inflammation. Among the key parameters to consider are intracellular ROS levels, temperature, mitochondrial dysfunction, apoptosis/necrosis, HSP70 expression, cytokine production, macrophage activation, and dendritic cell maturation. In summary, controlled phototoxicity can be viewed as a key principle of safety-by-design, aimed at maximizing ICG-related ROS and thermal effects in pathological tissues.

## 7 Safety, regulatory, and safety-by-design considerations for translating NIR-responsive nanomaterials

Safety assessment of NIR-responsive nanomaterials should include intrinsic nanotoxicity and NIR effects like photothermal heating, ROS generation, photochemical changes, and degradation. Key endpoints, cytotoxicity, apoptosis, oxidative stress, mitochondrial dysfunction, genotoxicity, hemocompatibility, and inflammation, must be measured with controls for nanomaterial, NIR, and both. Characterization of particle size, morphology, surface charge, aggregation, composition, and dose is crucial because these factors influence biological responses. Documents from organizations such as the OECD advocate for uniform testing to achieve a reliable safety assessment. The effectiveness of 2D cultures is limited; therefore, scientists have turned to 3D spheroids, organoids, organ-on-chips, and disease models to mimic tissue structure and gradients. Regulatory processes today depend on frameworks established by organizations such as the FDA, ISO, OECD, and ICH, rather than on direct protocols for NIR. The FDA provides information on particle size, form, makeup, clustering, stability, and constancy in production, thus assessing nanoparticles separately, but fails to consider parameters such as wavelength, irradiance level, thermal dose, oxygen dependence, and photodegradation. The limited clinical transferability of the photothermal NIR nanoparticle is illustrated by AuroShell®, which can be classified as a silica-gold-based nanoparticle used in NIR photothermal therapy. Even though this substance has undergone clinical testing, the use of nanoparticles in photothermal treatment is not considered common practice. This fact is due to the problems connected to the production, properties, penetration into tissues, light dosimetry, safety, distribution, and retention of nanomaterials (Buiel et al., 2025). The limitations highlight the importance of safety-by-design principles during material development, such as biodegradability, size/morphology, biocompatible surfaces, low residual chemicals, predictable degradation, and efficient clearance. NIR-absorbing agents need improvement to balance photothermal conversion, ROS generation, photostability, and toxicity. Nanoplatforms should minimize harmful degradation products. Using dosimetry, human models, pharmacokinetics, degradation profiling, and long-term toxicity is crucial for clinical use. Evaluating physical and chemical properties, safety, manufacturing quality, and irradiation parameters is essential for NIR nanomaterials. Despite guidelines from FDA, ISO, OECD, and ICH, traditional models have limitations, necessitating standardized evaluation of biodistribution, degradation, clearance, immunotoxicity, and phototoxicity. Reproducible manufacturing, batch consistency, stability, and device calibration are also necessary. Material safety and efficacy depend on composition, biological interactions, and irradiation conditions. Future research should focus on predictive toxicity, long-term safety, disease-specific activation, and advanced biological models.

## 8 Conclusion and future perspective

NIR-responsive nanomaterials offer promising opportunities for integrated imaging and therapy. Still, their clinical translation requires balancing therapeutic performance with biological safety. Material properties, biological interactions, degradation, and irradiation conditions collectively influence efficacy and toxicity. Future research should prioritize predictive structure–toxicity models, long-term safety assessment, and disease-specific activation strategies to minimize off-target effects. Integrating advanced biological models, artificial intelligence, and standardized irradiation protocols may further facilitate the development of safe and clinically translatable theranostic platforms.
